# Assessment of Volume Depletion in Children with Malaria

**DOI:** 10.1371/journal.pmed.0010018

**Published:** 2004-10-19

**Authors:** Timothy Planche, Myriam Onanga, Achim Schwenk, Arnaud Dzeing, Steffen Borrmann, Jean-François Faucher, Antony Wright, Les Bluck, Leigh Ward, Maryvonne Kombila, Peter G Kremsner, Sanjeev Krishna

**Affiliations:** 1Department of Cellular and Molecular Medicine, Infectious Diseases, St. George's Hospital Medical SchoolLondonUnited Kingdom; **2**Medical Research Unit, Albert Schweitzer HospitalLambarénéGabon; **3**Département de Parasitologie, Mycologie, et Médecine Tropicale, Faculté de Médecine, Université des Sciences de la SantéLibrevilleGabon; **4**Coleridge Unit, North Middlesex University HospitalLondonUnited Kingdom; **5**Department of Parasitology, Institute of Tropical Medicine, University of TübingenTübingenGermany; **6**Department of Biochemistry, University of QueenslandSt LuciaAustralia; **7**Elsie Widdowson Laboratory, Medical Research Council Human Nutrition ResearchCambridgeUnited Kingdom; Mahidol UniversityThailand

## Abstract

**Background:**

The degree of volume depletion in severe malaria is currently unknown, although knowledge of fluid compartment volumes can guide therapy. To assist management of severely ill children, and to test the hypothesis that volume changes in fluid compartments reflect disease severity, we measured body compartment volumes in Gabonese children with malaria.

**Methods and Findings:**

Total body water volume (TBW) and extracellular water volume (ECW) were estimated in children with severe or moderate malaria and in convalescence by tracer dilution with heavy water and bromide, respectively. Intracellular water volume (ICW) was derived from these parameters. Bioelectrical impedance analysis estimates of TBW and ECW were calibrated against dilution methods, and bioelectrical impedance analysis measurements were taken daily until discharge. Sixteen children had severe and 19 moderate malaria. Severe childhood malaria was associated with depletion of TBW (mean [SD] of 37 [33] ml/kg, or 6.7% [6.0%]) relative to measurement at discharge. This is defined as mild dehydration in other conditions. ECW measurements were normal on admission in children with severe malaria and did not rise in the first few days of admission. Volumes in different compartments (TBW, ECW, and ICW) were not related to hyperlactataemia or other clinical and laboratory markers of disease severity. Moderate malaria was not associated with a depletion of TBW.

**Conclusions:**

Significant hypovolaemia does not exacerbate complications of severe or moderate malaria. As rapid rehydration of children with malaria may have risks, we suggest that fluid replacement regimens should aim to correct fluid losses over 12–24 h.

## Abstract 2

Some investigators have suggested that children with malaria need a lot of fluids. But this paper casts doubt on this assumption, and assesses a novel way to assess fluid balance

## Introduction

Malaria claims one million lives annually, with more than 90% of these being those of children in sub-Saharan Africa [1]. Most deaths of hospitalised children occur in the first 24 h after admission. Even modest improvements in management during this time may improve survival [2]. There is considerable disagreement about the degree to which children with severe malaria become hypovolemic. In east African studies, clinical signs of severe malaria (such as tachycardia, prolonged capillary refill times, and decreased urine volume) have been interpreted as evidence for volume depletion [2,3,4,5,6]. However, determining fluid compartment volumes is the first and most critical step in optimising fluid replacement therapy for children with malaria because clinical assessment of fluid status is difficult and imprecise [7]. Our study was designed to measure total body water volume (TBW) and extracellular water volume (ECW) using nonradioactive tracer dilution techniques and to derive intracellular water volume (ICW). Bromide distributes in the extracellular space so that concentrations measured 2–4 h after administration safely and reliably estimate ECW. Heavy water (^2^H_2_O) space represents TBW. ICW is calculated by subtraction of the ECW from the TBW.

Tracer dilution methods are expensive and invasive and cannot be repeated at short intervals. Therefore, we simultaneously calibrated a noninvasive technique of bioelectrical impedance analysis (BIA) [8] to estimate the fluid volumes. BIA measures the opposition (impedance) of the body to the flow of a small alternating current between electrodes placed on the hand and the foot, and then estimates TBW and ECW using regression equations derived by calibration against ‘gold standard' tracer measurements of fluid volumes.

We hypothesised that volume changes in fluid compartments would reflect disease severity in malaria and that these changes would be related to established markers of disease severity [9]. We also calibrated BIA assessments in children with moderate and severe malaria with direct measurements of volume of TBW and ECW.

## Methods

The study was conducted at the Albert Schweitzer Hospital, Lambaréné, Gabon, and Centre Hospitalier de Libreville, Gabon. It was approved by the ethics committees of the International Foundation of the Albert Schweitzer Hospital, the Gabonese Ministry of Health, and the University of Tübingen.

Children (aged 1 to 10 y, inclusive) admitted with suspected severe or moderate malaria were referred to the study team, who assessed them within 15 min, and the children were admitted to the study once informed consent had been obtained from the parents. Malaria was defined as the presence of asexual forms of Plasmodium falciparum in thick or thin blood films. Severe malaria was malaria with one or more of the following features: blood lactate ≥ 5 mmol/l, blood glucose ≤ 2.2 mmol/l, Blantyre coma score ≤ 2, or repeated, observed seizures [2]. Moderate malaria was malaria without any of the features of severe malaria but with a requirement for parenteral treatment because of one or more of the following: a history of frequent (> 2) and recent vomiting (within 12 h), drowsiness, obtundation, or prostration [2]. Alternative diagnoses were excluded clinically.

### Assessment and Management

On admission children were weighed (undressed) with pediatric scales accurate to within 100 g (Seca, Birmingham, United Kingdom). A history was taken from parents, and the child was examined with particular attention to signs of dehydration, including: capillary refill time, skin turgor, sunken eyes, dry mucous membranes, and absence of tears. Vital signs, blood glucose and lactate concentrations, and hematocrit and parasitaemia were measured every 4 h for the first 24 h and then every 6 h until recovery. If peripheral venous access was impracticable, then a femoral central venous catheter was inserted, and the central venous pressure was measured every 4 h with a manometer zeroed at the midaxillary line.

Children were managed in a standard manner as previously described [10,11,12]. All received intravenous quinine (20 mg/kg salt intravenously as a loading dose given over 4 h, then 10 mg/kg intravenously every 12 h until able to take oral medication) (Quinimax, Sanofi Synthelabo, Paris, France). Hypoglycaemia (blood glucose ≤ 2.2 mmol/l) was treated with 25% glucose (2 ml/kg). Convulsions were treated with diazepam (0.3 mg/kg intravenously or 0.5 mg/kg intrarectally) (Roche, Basel, Switzerland), and repeated convulsions were treated with phenobarbital (7.5 mg/kg intramuscularly).

In addition to a 5% or 10% dextrose infusion (at least 3 mg/kg/min, i.e., 1.6 or 3.2 ml/kg/h), physicians were free to give any fluid replacement regimen as clinically indicated, including boluses of saline or blood. A strict fluid input/output chart was kept for each child. A blood transfusion of 20 ml/kg of cross-matched whole blood tested for blood-borne pathogens was given over 4 h if the hematocrit fell below 15%.

### Measurement of TBW and ECW

A sterile standard dosing solution was prepared by adding 119 ml of ^2^H_2_O per litre of 2.315% sodium bromide solution. A baseline sample of blood (3 ml) was drawn, and the plasma frozen at −70 °C for bromide and ^2^H_2_O assays. At the start of the study, 2.8 ml/kg of the dosing solution was administered intravenously over 20 min. Four hours after injection a second blood sample was drawn (1.5 ml) for the determination of blood bromide and ^2^H_2_O concentrations. Parents were asked to return with their children 28 d after admission; children were examined, and measurement of TBW and ECW repeated.

^2^H enrichment was measured in duplicate by isotope ratio mass spectrometry, using a Sira 10 instrument (Micromass, Cheshire, United Kingdom) as described [13]. The precision of the TBW determination was estimated at 0.3% of the value obtained. Batch analysis of bromide enrichment in plasma was performed by high-performance anion-exchange liquid chromatography as described [14]. The intra-assay coefficient of variation for bromide was better than 1.5%.

BIA was performed using a SEAC SFB3 multifrequency bioimpedance meter (Impedimed, Brisbane, Australia). An alternating electrical current of 200 μAmp was applied between 2 Ag/AgCl electrodes at the right hand and right foot. Whole body and segmental impedance were measured by rotating the sensing Ag/AgCl electrodes between four sites on the ankles or wrists as described [13]. Each set of measurements was taken at 496 frequencies between 4 kHz and 1012 kHz. Measurements were taken at 0 h, 4 h, 12 h, 24 h, and discharge. The process took about 2 min to perform and was simpler than obtaining an electrocardiogram. Data were analysed with software (Bioimp, version 1.1.0, Impedimed) that uses nonlinear regression to fit measured data to semicircular Cole-Cole plots [15] of reactance against resistance. Resistance and reactance values, obtained from the Cole-Cole plot for specific frequencies (0, 4, 50, and 100; characteristic and infinite kHz) were used in further analysis [13].

Plasma electrolytes were measured (Beckman Coulter, Allendale, New Jersey, United States), and osmolality was calculated using freezing point depression on a Micro Osmometer (Vitech Scientific, West Sussex, United Kingdom). Osmol gap was calculated as described [16]:



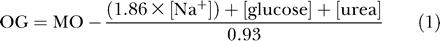



where OG is osmol gap, MO is measured osmolality, [Na*^+^*] is plasma sodium concentration (millimoles/litre), [glucose] is plasma glucose concentration (millimoles/litre), [urea] is plasma urea concentration (millimoles/litre), 1.86 is a correction factor as sodium chloride is only 93% dissociated, and 0.93 is the assumed proportion of water in plasma.

### Statistical Methods

Statistical analyses were carried out using Stata Statistical Software (Releases 6.0–8.0, College Station, Texas, United States). After checking distributions with the Shapiro-Wilks *W* test, and transforming data logarithmically if necessary, we analysed normally distributed data by two-tailed Student's *t* test and nonparametric data with the Wilcoxon sign-rank test. Proportions were compared with Fisher's exact test, and correlations were assessed by linear regression analysis of Pearson or Spearman. Predictive values for TBW and ECW from impedance measurements were obtained by multivariate analysis in a backward elimination process with *p <* 0.05 for entry and *p <* 0.10 for exit, confirmed by forward selection. This analysis was based on the first pair of BIA and isotope dilution measurements after admission. In a second step, whole body BIA models were replaced with segmental BIA models as previously described [13] and compared with whole body estimates. Errors in the BIA estimate and isotope dilution methods were compared. TBW estimates were corrected for fluid input and output during the period of measurement.

Sample size was calculated from previously published values [17,18] assuming a mean (SD) for convalescent TBW of 586 (30) ml/kg; we wished to detect a 5% difference in TBW between severe and moderate cases with a power of 90%.

## Results

Between October 1999 and March 2000, 205 children who were judged ill enough to be hospitalised were referred to the study team. One hundred and thirty-two children had malaria, 20 with severe and 35 with moderate disease. Ten children with moderate and two with severe malaria were ineligible for study because of their age, and for seven children (one with severe malaria) consent could not be obtained. One child died before inclusion into the study, leaving nineteen children with moderate and sixteen with severe malaria admitted to this study. The median (interquartile range [IQR]) time from admission to administration of tracers was 54 (37–84) min, during which complications such as convulsions or hypoglycaemia were treated. The baseline characteristics of children are given in Table 1. Those with severe malaria had significantly higher pulse rates, mean arterial pressure and blood lactate concentrations, and a longer capillary refill time (*p <* 0.001), compared to children with moderate malaria. Capillary refill time and blood lactate concentrations were correlated with each other (adjusted r^2^ [adj r^2^] = 0.25, *p =* 0.031).

**Table 1:**
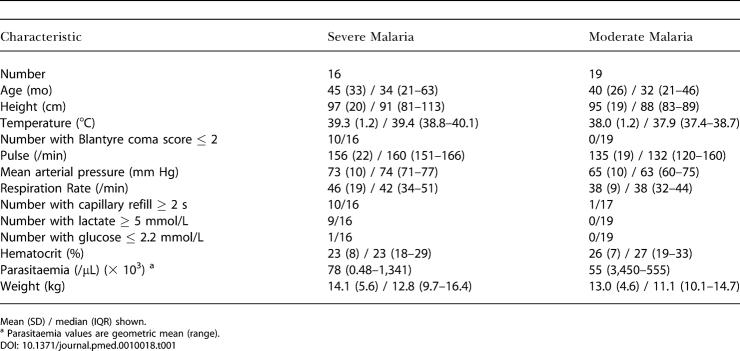
Baseline Characteristics of Children with Malaria

Mean (SD) / median (IQR) shown

^a^ Parasitaemia values are geometric mean (range)

**Figure 1:**
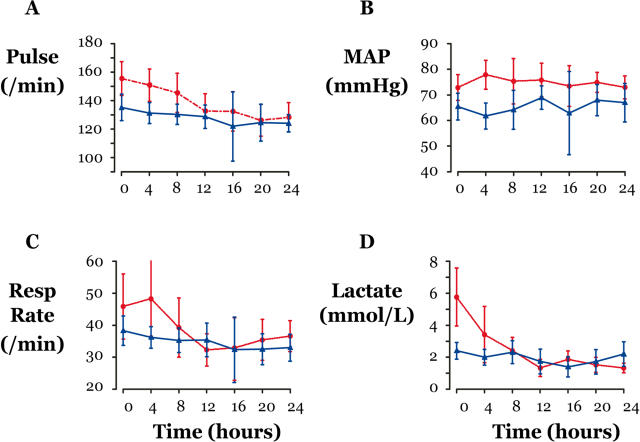
Vital Signs of Children during the First 24 h after Admission Mean and 95% confidence interval shown. Red circles, severe malaria; blue triangles, moderate malaria. (A) Pulse (per minute), (B) mean arterial pressure (millimetres Hg), (C) respiratory rate (per minute), and (D) blood lactate concentration (millimoles/litre).

The volume of fluid (including blood) given in the first 4 h of the study was similar in children with severe (median [IQR, range] of 3.7 [2.2–5.8, 2.1–11.4] ml/kg/h) and moderate (median [IQR, range] of 3.3 [2.2–4.2, 1.2–15.1] ml/kg/h) malaria (*p* > 0.5). There was rapid correction of vital signs in all children (Figure 1), and vital signs were similar in both study groups by 8 h. There were two deaths (4.5 and 6 h after admission), and two children with severe malaria had major persistent neurological deficits (4/16 [25%] with adverse outcomes). Central venous pressure (CVP) measurements were obtained in six children with severe malaria. The median (IQR) CVP on admission was +6.5 (3–7.5) cm H_2_O with no significant rise in the first 24 h.

### Fluid Volumes

Volume determinations using isotope dilution were available at baseline in all but one of the children with severe malaria. The values for the TBW, ECW, and ICW are given in Table 2. The TBW was significantly lower at admission compared with day 28 for the severe cases (*p =* 0.028) but not for moderate cases (*p =* 0.109). The mean (SD) TBW was lower in severe than moderate malaria at admission: 524 (44) ml/kg versus 555 (50) ml/kg (*p =* 0.052). The mean (SD) change in TBW between admission and follow up was 48 (42) ml/kg and 12 (37) ml/kg for severe and moderate cases, respectively. Individual data from the children with severe malaria are shown in Table 3.

**Table 2:**
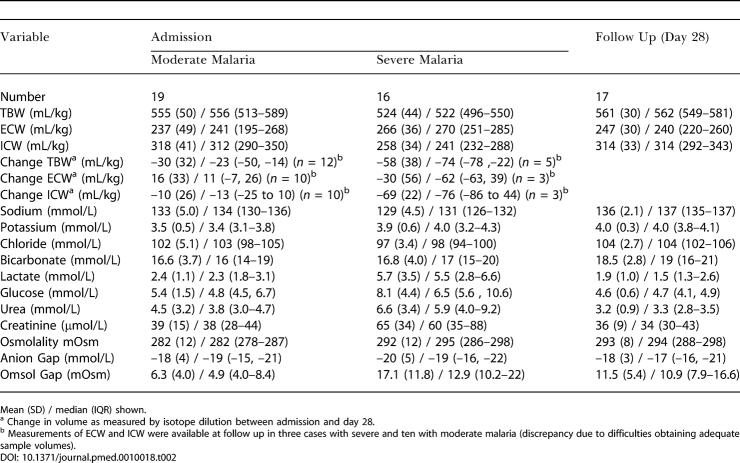
Volumes Determined by Tracer Dilution and Electrolyte Measurements

Mean (SD) / median (IQR) shown

^a^ Change in volume as measured by isotope dilution between admission and day 28

^b^ Measurements of ECW and ICW were available at follow up in three cases with severe and ten with moderate malaria (discrepancy due to difficulties obtaining adequate sample volumes)

**Table 3:**
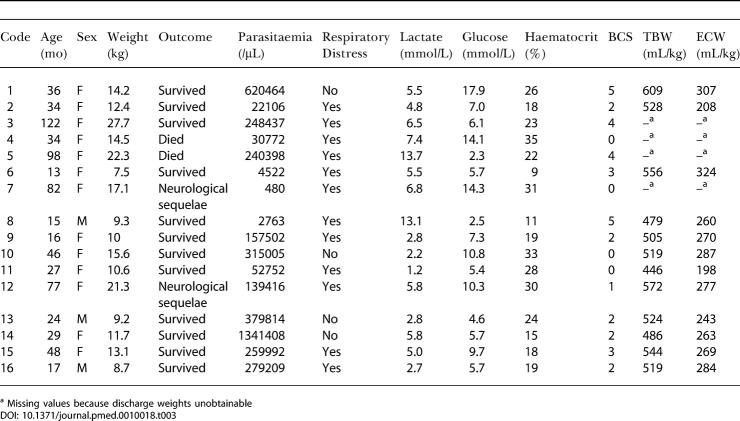
Individual Details of Severely Ill Children

^a^ Missing values because discharge weights unobtainable

Bromide space (ECW) measurements were available in all but two children with severe malaria and in all but one of the children with moderate malaria. At baseline, the mean (SD) ECW was significantly lower in children with moderate than in those with severe malaria (*p =* 0.045). Admission ICW measurements were significantly lower in children with severe malaria than in those with moderate malaria (*p <* 0.001).

### Bioelectrical Impedance Analysis

As predicted by theory [8], there was a strong correlation between height^2^ divided by impedance at 50 kHz (*H* ^2^/*Z*_50_) and measured TBW. The ‘best fit' regression equation to predict TBW from BIA was derived using the variables age, weight, and *H* ^2^/*Z*_50_ (standard error of the estimate [SEE] = 0.435, adj r^2^ = 0.975):



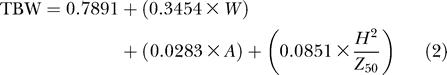



where *A* is age (months), *H* is height (centimetres), *W* is weight (kilograms), and *Z*_50_ is impedance at 50 KHz frequency. Disease severity and gender did not contribute significantly to this model, allowing data from admission to be pooled in this prediction equation. Repeating the analysis using impedances measured at other frequencies (4–1012 kHz) did not show a clear advantage, so all data for TBW prediction are given for measurement at 50 kHz.

By contrast, ECW was not significantly associated with age or weight, and in agreement with previous studies in babies [19] *H* ^2^/*R*_0_ emerged as the strongest predictive term (SEE = 0.584, adj r^2^ = 0.753):



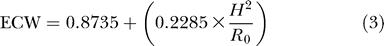



where *H* is height (centimetres), and *R_0_* is resistance at zero frequency. Again, disease severity and gender did not contribute significantly to this model. Prediction equations based on segmental BIA data for ECW and TBW were inferior to whole body BIA models and are therefore not shown here.

**Figure 2:**
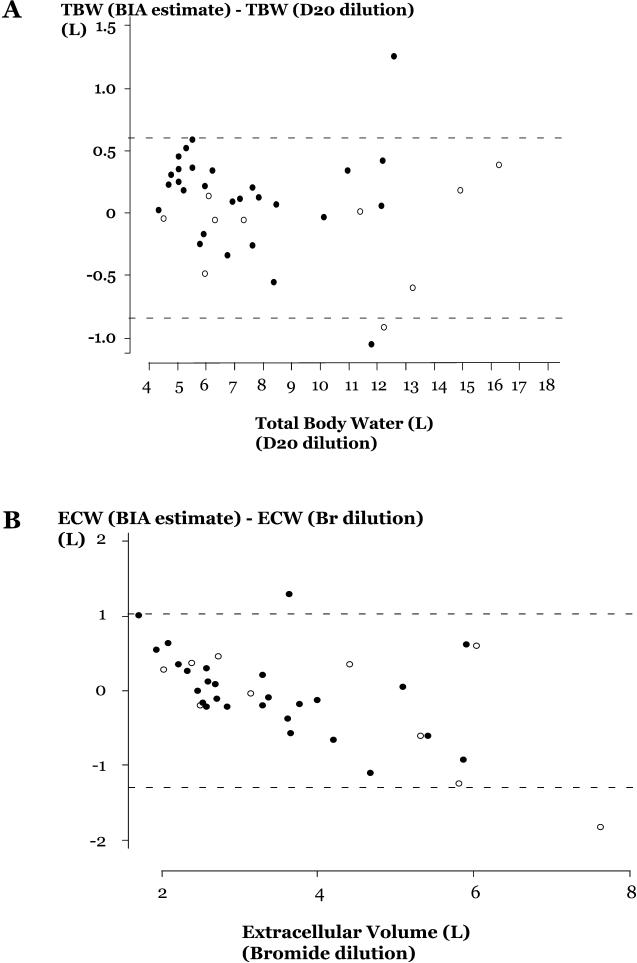
Plots of TBW and ECW Estimates from Isotope Dilution and BIA Calculation with Measured Values Filled circles, admission value; open circles, day 28 follow up value; dotted lines, 95% confidence intervals for values. (A) TBW and (B) ECW. D_2_O, heavy water.

Errors in BIA estimates of TBW and ECW compared to values measured by isotope dilution are displayed with 95% limits of agreement in Figure 2A and 2B, respectively.

### Fluid Volumes from BIA

**Figure 3:**
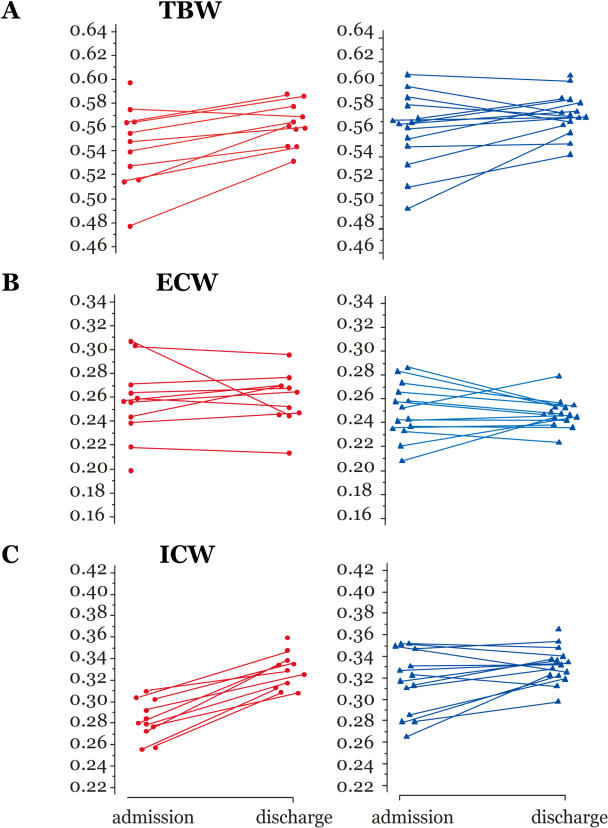
Body Fluid Compartment Volumes Derived from BIA on Admission and Discharge Red circles, severe malaria; blue triangles, moderate malaria. (A) TBW (litres/kilogram), (B) ECW (litres/kilogram), and (C) ICW (litres/kilogram).

Fluid volume estimates were available from BIA in 14 children with moderate and 11 with severe malaria on admission and 16 with moderate and 12 with severe malaria at discharge. Values for the TBW, ECW, and ICW are shown in Figure 3.

BIA-determined TBW was significantly lower in those with severe compared with moderate malaria (mean [SD] of 539 [32] versus 562 [30] ml/kg, *p =* 0.034). TBW increased from admission to discharge in children with severe malaria by 37 (33) ml/kg or 6.7% (6.0%) (paired *t* test, *p =* 0.012), but there were no significant changes in TBW for children with moderate malaria between admission and discharge.

ECW did not differ between the severe and moderate groups on admission and did not change significantly between admission and discharge. Serial measurements of ECW did not show any rise during the first 4 d after admission, at discharge, or at day 28 in children with severe or moderate malaria (data not shown). ICW at admission was significantly lower in the severe malaria group than in the moderate malaria group, with a mean (SD) of 293 (17) ml/kg versus 325 (28) ml/kg (*p =* 0.002). ICW remained unchanged between admission and discharge in children with moderate malaria. In the severe malaria group mean (SD) of ICW at admission was 40 (22) ml/kg, or 11.7% (11.0%) lower at admission than at discharge (*p =* 0.002). There were no differences in the fluid volume measurements of the four children with adverse and those with good outcomes.

There were no relationships found between TBW, ECW, or ICW, or changes in TBW, ECW, and ICW between baseline and discharge, and any of the following markers of severity in malaria: blood lactate concentration, coma score, plasma creatinine concentration, peripheral parasitaemia, blood glucose, coma recovery time, time to walk, time to eat, time to drink, length of hospital stay, or having a history of diarrhoea or vomiting.

Admission weight relative to the weight at discharge showed a mean (SD)/median (IQR, range) percentage deficit of −4.3% (4.4%)/−4.0% (−1.3% to −6.1%, −0.1% to −11.0%) for the children with severe malaria on admission (*p =* 0.002). For those with moderate malaria the deficit was not significant: −0.3% (4.6%) /−0.9% (1.6% to −1.5%, 7.0% to −8.1%) (*p =* 0.41). As predicted, the deficits in weight and TBW were correlated (adj r^2^ = 0.67, *p <* 0.001).

### Electrolyte Measurements

The concentrations of plasma electrolytes are shown in Table 2. The mean (SD) concentrations of sodium were significantly lower for the children with severe malaria than for those with moderate malaria (*p =* 0.036). Plasma potassium concentration and osmolality were higher in children with severe than in those with moderate malaria (*p =* 0.039 and *p =* 0.021, respectively). Plasma urea and creatinine were significantly higher in those with severe malaria. Osmol gap was significantly higher in children with severe than in those with moderate malaria (*p <* 0.001), with 15/16 children in the severe group having an osmol gap greater than 8.2 mOsm (considered high in United States children [20]).

## Discussion

We have shown that severe childhood malaria is associated with mild dehydration in most cases, with a mean (SD) depletion of TBW of 37 (33) ml/kg, or 6.7% (6.0%). Only 3/16 children (19%) in our study had moderate volume depletion (> 60–90 ml/kg), and none were severely dehydrated (> 100 ml/kg) [21]. Moderate malaria was not associated with any significant changes in TBW. Consistent with a lower TBW in severe disease, ICW was depleted in children with severe malaria by a mean (SD) of 40 (22) ml/kg, an 11.7% (11.0%) difference. However, we found no relationship between TBW, ECW, and ICW and clinical and laboratory markers of disease severity, in particular the two most important prognostic indicators of fatal outcome: hyperlactataemia and Blantyre coma score [9].

Our findings suggest that the degree of dehydration in children with severe malaria is unlikely to be a primary pathological process in the evolution of the disease. These findings are also consistent with our previous suggestion that hyperlactataemia arises from tissue hypoxia resulting from microvascular obstruction by infected erythrocytes [12,22] rather than gross hypovolaemia. ECW did not change significantly during hospitalisation. Furthermore, our fluid replacement regimen (median [IQR] 3.7 [2.2–5.8] ml/kg/h) normalised vital signs and blood lactate within 8 to 12 h.

Our estimates of fluid compartment volumes in children after recovery from malaria are entirely consistent with previous work in children (range: TBW, 540–640 ml/kg; ECW, 250–320 ml/kg; ICW, 260–340 ml/kg) [17], although our study is the first that we know of to examine fluid status in childhood malaria. Studies in adults with uncomplicated or moderate malaria have given conflicting results [23,24,25,26,27].

In sepsis, ECW increases by up to 50% of TBW because capillary permeability increases by up to 300% of normal. There are no significant increases in ECW in children with severe malaria, confirming earlier studies that indicate that sepsis and malaria syndromes result from different pathophysiological processes [9,28]. Studies on fluorescein angiography in children and adults also confirm there is no increased capillary permeability in severe malaria [23,29]. Furthermore, CVP measurements in a subgroup (6/16) of children were not low, and did not change significantly after 24 h of intravenous fluid replacement. Taken together, these findings do not suggest that volume depletion or increased capillary permeability are important to the pathophysiology of malaria in our population.

Hyponatraemia [30] has been attributed to high and possibly inappropriate arginine vasopressin secretion [31] in severe malaria. Our findings (high osmol gap, low ICW, and normal ECW) are more in keeping with sick cell syndrome than with inappropriate arginine vasopressin secretion [32,33]. To conclude that arginine vasopressin is inappropriately elevated, renal function must be normal and volume depletion excluded. No severely ill child in this study fulfilled these criteria.

What are the implications of our findings for optimal fluid replacement therapy in malaria? We cannot answer precisely on the basis of measuring fluid compartment volumes because regimens are sometimes designed not only to correct existing fluid deficits and to provide maintenance requirements, but also to rehydrate more vigorously to maintain circulating volume. Such approaches are advocated by others for different populations of children with severe malaria, for example, in a series of studies published from Kilifi, Kenya [4,5,34]. However, estimates of fluid requirements for children with severe malaria have been based upon indirect measurements (such as monitoring vital signs and degree of acidosis) that are potentially misleading because they do not relate to the degree of fluid loss that we measured. Furthermore, adequately powered controlled studies aimed at defining appropriate fluid regimens for severe malaria are lacking, but should take into account our findings as well as the BIA methodology that we have now calibrated to measure fluid compartment volumes in malaria. Indeed, a fluid (0.9% saline) replacement rate of up to 20 ml/kg in 1 h is a considerably faster rate than we can advocate on the basis of our findings. In any case, BIA can now be used (equations 2 and 3) to measure ECW and TBW noninvasively to guide treatment in patients with severe malaria.

There are risks to over-vigorous fluid administration just as there are with inadequate fluid replacement, particularly in hospitals where measuring plasma electrolyte concentrations and providing assisted ventilation are difficult. These risks include pulmonary [3] and cerebral oedema [6] (which occur in adults and children, respectively) and dangerously rapid changes in plasma electrolyte concentrations. Children with severe malaria have a low ICW and are at risk of hypokalaemia if ICW is restored rapidly (< 4 h), particularly when there is relative hyperinsulinaemia due to quinine administration [12].

A correlation between capillary refill time and blood lactate concentrations but none with fluid volume status suggests that prolongation of capillary refill time may be due to the common underlying process of microvascular obstruction.

Our findings do not support the widespread use of aggressive fluid volume replacement in children with severe malaria. Clearly, volume depletion indicated by hypotension or by CVP measurements requires more aggressive therapy, but wherever possible plasma electrolyte concentrations should be closely monitored. Because of elevated requirements for glucose in childhood severe malaria (3–6 mg/kg/min) [35], there is a need to provide a maintenance fluid replacement rate of about 3 ml/kg/h [7,36]. In addition, fluid regimens should aim to replace mild fluid deficit within the first 12 to 24 h of admission.

The tracer dilution techniques that we have used are expensive and time-consuming and consequently not amenable to large-scale deployment. We took this opportunity to calibrate a much simpler methodology (BIA) to derive TBW and ECW and validated BIA estimations in this population. We are now using BIA measurements to assess much larger numbers of patients (J. Jarvis and S. K., unpublished data). BIA is an excellent noninvasive screening tool that should detect subgroups of children with severe malaria who may be severely volume depleted.

**Patient Summary**

**Background**

Although we have known for many years what causes malaria, how it is passed from person to person by mosquito, and how to treat the infection, more than a million people still die of malaria every year, mostly children under five years living in Africa. Children are affected most because they have not had the chance to develop the resistance to malaria that normally builds up over a lifetime when living in places with malaria. As well as being given specific drug treatments against the malaria, children are also often given fluids into their veins, as they appear dehydrated.

**What Did the Researchers Find?**

The researchers studied children who were sick with malaria and measured how dehydrated the children were. To do the measurements, they used an accepted technique that required injections into the vein, and also a newer, simpler method that used electrodes. Neither technique suggested that any of the children were severely dehydrated.

**What Does This Mean for Patients?**

The study suggests that severe dehydration isn't a big problem in children with severe malaria. So it may not be necessary to give lots of fluids into the vein. (While treatment of malaria is still being worked out, malaria can, of course, often be prevented by using insecticide-treated bednets.)

**Are There Any Problems with the Study?**

The new technique for measuring dehydration will need to be assessed in larger studies: this study is small, so the results may not be entirely accurate.

**Where Can I Get More Information?**

The World Health Organization is coordinating many of the initiatives to combat malaria (http://www.who.int/topics/malaria/en/).

Medicines for Malaria Venture is trying to develop new affordable antimalarial drugs (http://www.mmv.org/pages/page_main.htm).

## Abbreviations

^2^H_2_O: heavy water
adj r^2^: adjusted r^2^
BIA: bioelectrical impedance analysis
CVP: central venous pressure
ECW: extracellular water volume
ICW: intracellular water volume
IQR: interquartile range
SEE: standard error of the estimate
TBW: total body water volume
